# Adaptive fuzzy control for tendon-sheath actuated bending-tip system with unknown friction for robotic flexible endoscope

**DOI:** 10.3389/fnins.2024.1330634

**Published:** 2024-03-26

**Authors:** Fan Ren, Xiangyu Wang, Ningbo Yu, Jianda Han

**Affiliations:** ^1^The Tianjin Key Laboratory of Intelligent Robotics, College of Artificial Intelligence, Institute of Robotics and Automatic Information Systems, Nankai University, Tianjin, China; ^2^The Institute of Intelligence Technology and Robotic Systems, Shenzhen Research Institute of Nankai University, Shenzhen, China

**Keywords:** tendon-sheath mechanism, fuzzy control, friction compensation, robust control, robotic flexible endoscope

## Abstract

**Introduction:**

The tendon-sheath actuated bending-tip system (TAB) has been widely applied to long-distance transmission scenes due to its high maneuverability, safety, and compliance, such as in exoskeleton robots, rescue robots, and surgical robots design. Due to the suitability of operation in a narrow or tortuous environment, TAB has demonstrated great application potential in the area of minimally invasive surgery. However, TAB involves highly non-linear behavior due to hysteresis, creepage, and non-linear friction existing on the tendon routing, which is an enormous challenge for accurate control.

**Methods:**

Considering the difficulties in the precise modeling of non-linearity friction, this paper proposes a novel fuzzy control scheme for the Euler-Lagrange dynamics model of TAB for achieving tracking performance and providing accurate friction compensation. Finally, the asymptotic stability of the closed-loop system is proved theoretically and the effectiveness of the controller is verified by numerical simulation carried out in MATLAB/Simulink.

**Results:**

The desired angle can be reached quickly within 3 s by adopting the proposed controller without overshoot or oscillation in Tracking Experiment, demonstrating the regulation performance of the proposed control scheme. The proposed method still achieves the desired trajectory rapidly and accurately without steady-state errors in Varying-friction Experiment. The angle errors generated by external disturbances are < 1 deg under the proposed controller, which returns to zero in 2 s in Anti-disturbance Experiment. In contrast, comparative controllers take more time to be steady and are accompanied by oscillating and residual errors in all experiments.

**Discussion:**

The proposed method is model-free control and has no strict requirement for the dynamics model and friction model. It is proved that advanced tracking performance and real-time response can be guaranteed under the presence of unknown bounded non-linear friction and time-varying non-linear dynamics.

## 1 Introduction

**Figure 1:**
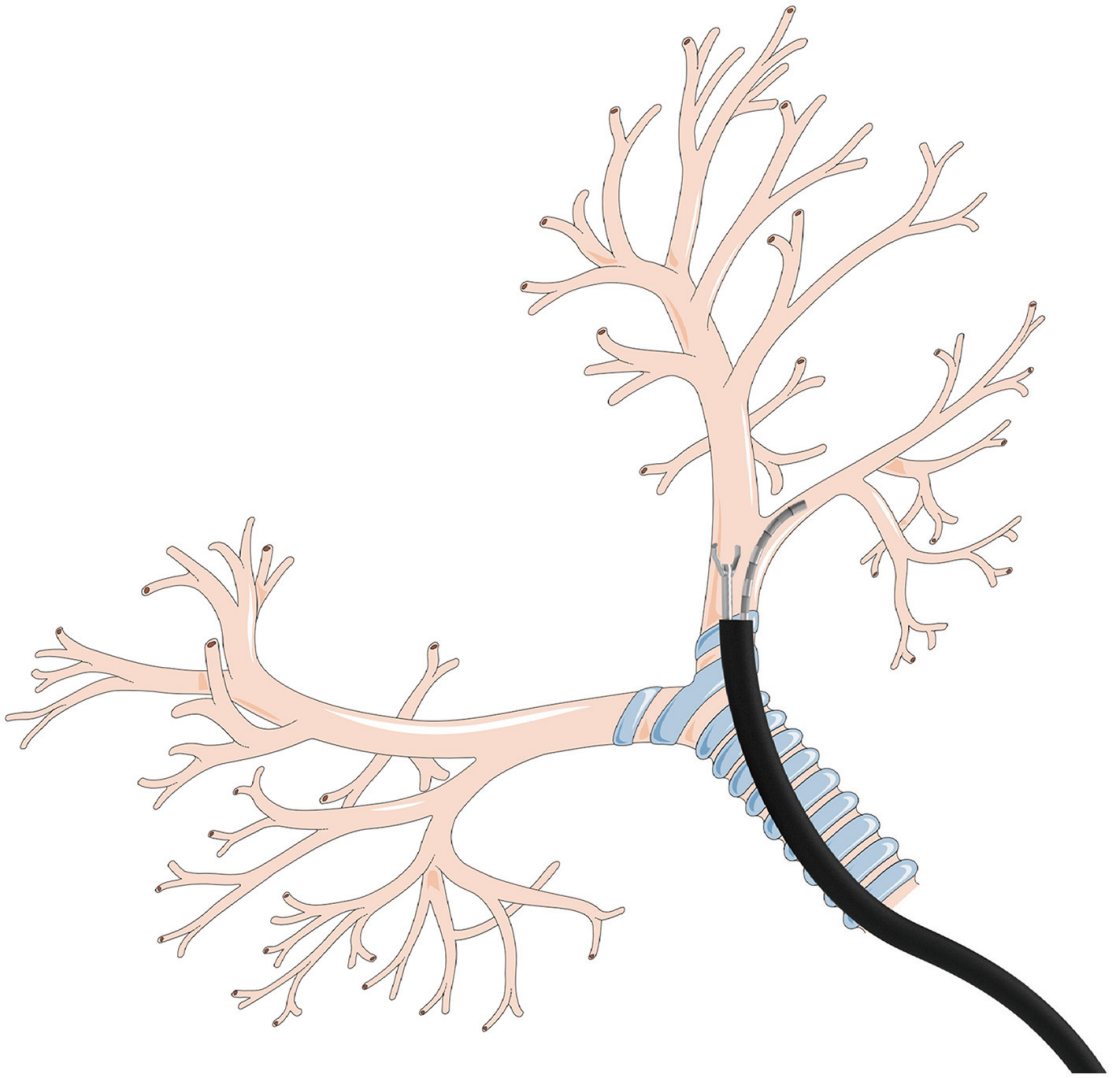
Illustration of TAB utilized in flexible bronchoscopy.

The tendon-sheath mechanisms (TSM) have attracted widespread interest in the field of surgery, pipeline inspection, and rehabilitation due to their flexibility, safety, and dexterity, such as applied in the neurosurgery surgical robot, the otolaryngology robot, the cardiac surgical robot, etc. (Burgner-Kahrs et al., 2015; Do et al., 2015a, 2016; Kang et al., 2020; Yin et al., 2020; Wang et al., 2023). The flexible characteristics of TSM make it highly versatile and applicable in narrow scenarios (Berthet-Rayne et al., 2018; Wang et al., 2020, 2021; Rho et al., 2021). Consequentially, numerous studies have focused on the application of TSM in the field of tendon-sheath actuated bending-tip (TAB) systems for natural orifice transluminal endoscopic surgeries (NOTES) as shown in Figure 1 in which bronchi is provided by Servier Medical Art (https://smart.servier.com) under CC BY 3.0 license. Due to the fact that the TAB can be deformed to fit the shape of the channel to avoid damaging the inner organ. However, the unknown friction existing in the tendon routing introduces motion backlash and persistent residual errors, which in turn increases the difficulties in the accurate tracking task of TAB systems. Therefore, it still requires extra effort to address the control issues of TAB systems.

The hysteresis effect is a crucial issue for the precise control of TAB systems. Most previous studies focused on improving the model accuracy of the hysteresis phenomenon in the TSM. For example, Do et al. (2015a,c, 2016) modified the Coleman-Hodgdon model for the hysteresis phenomenon of TSM and proposed a series of control methods, where no exact value of model parameters are required. Moreover, Thai et al. (2021) developed a simplified hysteresis model based on the generalized Bouc-Wen hysteresis model, while providing higher accuracy than previous Bouc-Wen models. Legrand et al. (2018) and Zhang et al. (2014) built piecewise models for the TAB systems. Similarly, Lee et al. (2021) proposed a simplified piecewise linear model to construct both backlash hysteresis and dead zone compensators together. Their research suggested that the errors from backlash hysteresis and dead zone are considerably reduced and therefore the accuracy of robotic control is improved.

Friction in the tendon-sheath actuator brings significant effects on the control performance of the objective system, such as additional chattering and input backlash. Previous research has focused on developing specific friction models for TSM to improve the modeling precision of the TAB. For example, inspired by the static Coulomb friction model and Hill muscle model, Zhang et al. (2017) presented a high-accuracy transmission model for the TSM. Moreover, Do et al. (2015b) developed a dynamic friction model to predict friction force for small displacement by using the LuGre model, which can capture the asymmetric loops and dead zone accurately. With consideration of the interaction continuity, Norouzi-Ghazbi and Janabi-Sharifi (2020) successfully proposed an equivalent model to estimate forces and moments applied to the sheath of the TSM. Furthermore, Jung and Bae (2016) proposed a feedforward scheme based on the force transmission model to compensate for the friction in a double-tendon-sheath actuated system. However, the TAB system has unknown dynamics and time-varying parameters, and it is very difficult to compensate for friction in real time precisely. In addition, whenever there is a change in working conditions caused by variations in TSM/TAB configurations, the friction model needs to be reidentified to achieve optimal results, which is challenging to apply in dynamic environments. Hence, there still remain open issues with design controllers that can overcome these model uncertainties for TAB systems.

Various control schemes have been proposed for TAB systems based on the concept of hysteresis compensation for TSM (Wu et al., 2014). Considerable works have focused on the perfect cancellation of backlash-like hysteresis using nonlinear adaptive algorithms and machine learning. Nguyen et al. (2014) developed an adaptive control scheme without requiring prior information of TAB systems to eliminate the hysteresis effects. To realize the position control of the TAB system Without sensory feedback, Wang et al. (2018) proposed a non-collocated position control method based on the inverse model of the TSM and 3-D reconstruction algorithm. Besides, Jiang et al. (2015) provided an adaptive PID controller with friction compensation for accurate position control of the dynamic model of TSM. Machine learning has also demonstrated its effectiveness in position control of the TAB system in recent years. For example, based on the kinematic model, Porto et al. (2019) proposed a learning-based hysteresis compensation technique, which directly employed the off-line parameters. Furthermore, Wu et al. (2019) proposed a neural-network-based sliding-mode control scheme by applying the radial basis function network to improve the position-control accuracy of the TAB system with modeling uncertainties and external disturbances. It is noteworthy that the integration of visual servoing with neural networks has recently become a research hotspot (Huang et al., 2022; Li et al., 2023; Cui et al., 2024). To ensure the tip of an instrument remains consistently centered in the camera, Huang et al. (2024) proposed an error learning-based sliding mode control, realizing the 4-DOF visual servo control in the robotic flexible endoscope system. The aforementioned research is impressive and offers a novel research perspective. However, these control schemes rely on an accurate reference model, and the updated process may be time-consuming in training. Therefore, developing a model-free adaptive controller to handle unknown friction for the TAB system is a worthwhile study.

In this paper, to achieve robust control of the TAB system with time-varying dynamics model parameters, a novel adaptive fuzzy controller is proposed. In particular, the complex frictional force between the tendon and sheath may be affected by the sheath deformation and lumina pressure, which brings difficulties to the accurate modeling of the TAB system. To handle this issue, the fuzzy logic system is utilized to compensate for the time-varying dynamics associated with unknown friction. The stability of the closed-loop system is guaranteed by Lyapunov-based analysis. Then, numerical simulations are implemented to further validate the tracking performance of the proposed control scheme. The main contributions of this manuscript can be summarized as follows:

1. By lumping the unknown friction, a novel dynamics model of TAB is established. It's worth mentioning that the proposed model diminishes its reliance on a priori information regarding friction and obviates the necessity for model-based linearization or supplementary linear parameterization conditions for global dynamics, thus rendering it more versatile.
2. To the best of our knowledge, this paper *first* applies fuzzy logic in the estimation of nonlinear dynamics due to unknown friction in TAB. By combining robust control with fuzzy logic regulated by an adaptive method, satisfactory performance can still be achieved under unknown time-varying friction and external perturbations.
3. The asymptotic stability of the closed-loop system is rigorously proven stable using the Lyapunov method, and the effectiveness and robustness of the controller under perturbations are verified through simulation experiments.

The rest of this paper is arranged as follows: Section 2 introduces the problem formulation including the dynamics model of the TAB system and control objective. Next, A robust fuzzy-based controller is given in Section 3 with the Lyapunov stability analysis in Section 4. Then, Section 5 describes the simulation results and analysis of the proposed method. Finally, Section 6 provides the conclusion of this research.

## 2 Problem formulation

**Figure 2:**
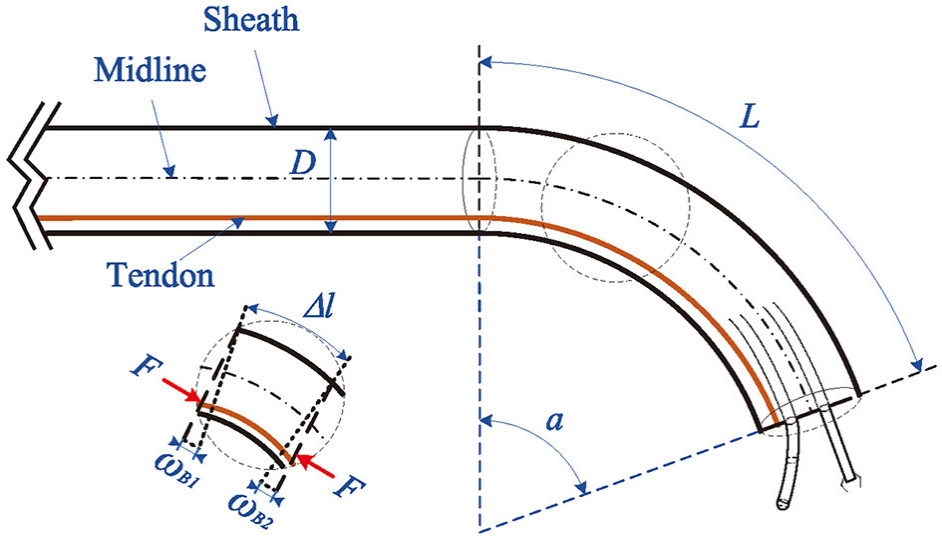
The driving principle of TAB system.

In this paper, the control problem for the bending motion of the TAB system was focused on. As shown in Figure 2, TAB consists of two sections of soft materials with different elastic modulus, and two symmetrically distributed actuators are built in as tendons. To facilitate the analysis of the dynamics, the bending tip is assumed to be a part of a constant curvature arc. Then, based on the Lagrangian theory, the dynamics model of the TAB system can be depicted as the following form as studied in Wang et al. (2023):


(1) $$\begin{array}{l} M\left(\alpha \right)\ddot{\alpha }+C\left(\alpha ,\dot{\alpha }\right)\dot{\alpha }+G\left(\alpha \right)=\mu -F_{f} \end{array}$$

where *M*(α), $C\left(\alpha ,\dot{\alpha }\right)$, *G*(α) represent the inertia matrix, centripetal and Coriolis force, and the gravitational force, respectively. The detailed physical meaning of parameters in Equation (2) are given in Table 1. μ represents the input control, *F*_*f*_ denotes the unknown friction. *M*(α), $C\left(\alpha ,\dot{\alpha }\right)$, *G*(α) in Equation (1) are depicted, respectively, as follows:


(2) $$\begin{array}{l} M\left(\alpha \right)=\frac{mL_{f}^{2}}{3\alpha^{2}},\;C\left(\alpha ,\dot{\alpha }\right)=-\frac{mL_{f}^{2}\dot{\alpha }}{3\alpha^{3}},\;G\left(\alpha \right)=\frac{5\pi ED^{4}\alpha }{64L_{f}} \end{array}$$

where α, $\dot{\alpha }$, *L*_*f*_, *D*, *m*, and *E* represent the bending angle, the bending angular velocity, the length, the diameter, the mass of the bending part, and Young's modulus of the scope's epidermis, respectively. For simplicity, the term *M*(α), $C\left(\alpha ,\dot{\alpha }\right)$, and *G*(α) will be abbreviated as *M*, *C* and *G* respectively. According to Equation 2, it can be found that they satisfy the following properties.

**Table 1:** Nomenclature and symbols in nominal dynamics model.

**Symbols**	**Physical meaning**	**Unit**
α	Bending angle of TAB's bending part	deg
α_*d*_	Desired angle	deg
*m*	Mass of TAB's bending part	kg
*L*_*f*_	Length of TAB's bending part	m
*E*	Young's modulus of bending part's epidermis of TAB	Pa
*D*	Diameter of TAB's bending part	m

*Property 1*. In this paper, α is the system output, and *M*(α) is positive and bounded, which can be described as follows:


(3) $$\begin{array}{l} \exists x_{1}>0,x_{2}>0,x_{1}\leq M\left(\alpha \right)\leq x_{2} \end{array}$$

*Property 2*. In Equation 2, *M*(α) and $C\left(\alpha ,\dot{\alpha }\right)$ are associated and satisfy


(4) $$\begin{array}{l} \forall \alpha \in R^{n},\frac{1}{2}\dot{M}\left(\alpha \right)-C\left(\alpha ,\dot{\alpha }\right)=0 \end{array}$$

It is generally accepted that certain models take major friction effects into account, notably encompassing Coulomb friction and Stribeck effects in tandem, with the aim of achieving a more precise representation of friction. Regrettably, owing to the material creepage and the time-varying contact force between the tendon and the sheath, attaining a precise description of friction distribution in TAB solely reliant on existing models is a formidable challenge (Zhang et al., 2017; Norouzi-Ghazbi and Janabi-Sharifi, 2020; Yin et al., 2022). Moreover, the static controller struggles to effectively handle dynamic uncertainties caused by the bending of soft material. In practical application, the time-varying friction between the tendon and sheath is typically assumed to change slowly and without abrupt variations. Nevertheless, the friction *F*_*f*_ and model parameters *M*(α), $C\left(\alpha ,\dot{\alpha }\right)$, *G*(α) are also unknown but bounded (UBB). The basic control task is to estimate dynamic parameters in real-time to achieve the tracking control of the bending part under the disturbances of unknown friction. Specifically, the control task can be quantified as follows:


(5) $$\begin{array}{l} \underset{t\to \infty }{\lim}e\left(t\right)=\underset{t\to \infty }{\lim}\alpha \left(t\right)-\alpha_{d}=0 \end{array}$$

where α_*d*_ represents the desired angle.

## 3 Controller design

An angle control algorithm based on fuzzy adaptive sliding mode control is presented in this paper. In order to facilitate the subsequent procedure, from Equation 5, the angle tracking errors, angular velocity tracking errors, and angular acceleration tracking errors of the bending part are defined respectively as follows:


(6) $$\begin{array}{l} e=\alpha_{d}-\alpha \\ \dot{e}={\dot{\alpha }}_{d}-\dot{\alpha }\\ \ddot{e}={\ddot{\alpha }}_{d}-\ddot{\alpha } \end{array}$$

where $\ddot{\alpha }$, α_*d*_, ${\dot{\alpha }}_{d}$ and ${\ddot{\alpha }}_{d}$ represent the angular acceleration, desired angle, angular velocity, and angular acceleration, respectively. An intermediate variable ${\dot{\alpha }}_{a}$ is defined as follows:


(7) $$\begin{array}{l} {\dot{\alpha }}_{a}={\dot{\alpha }}_{d}+c\left(\alpha_{d}-\alpha \right) \end{array}$$

where *c* is a known positive constant. From Equations 6 and 7, the sliding surface is defined as follows:


(8) $$\begin{array}{l} s=\dot{e}+ce={\dot{\alpha }}_{a}-\dot{\alpha } \end{array}$$

Then, the derivative of the sliding mode variable is derived as follows:


(9) $$\begin{array}{l} \dot{s}=\ddot{e}+c\dot{e}={\ddot{\alpha }}_{a}-\ddot{\alpha } \end{array}$$

From Equation 1, the dynamics model can be written as follows:


(10) $$\begin{array}{l} \ddot{\alpha }=M^{-1}\left(\mu -C\dot{\alpha }-G-F_{f}\right) \end{array}$$

From Equations 8–10, it can be presented as follows:


(11) $$\begin{array}{l} M\dot{s}=M{\ddot{\alpha }}_{a}-M\ddot{\alpha }\\ \;=M{\ddot{\alpha }}_{a}-\mu +C\dot{\alpha }+G+F_{f}\\ \;=-Cs-\mu +F_{f}+M{\ddot{\alpha }}_{a}+C{\dot{\alpha }}_{a}+G \end{array}$$

The unknown dynamics in Equation 11 is defined as a nonlinear function $P=M{\ddot{\alpha }}_{a}+C{\dot{\alpha }}_{a}+G$. Then, it can be simplified that


(12) $$\begin{array}{l} {\ddot{\alpha }}_{a}=M^{-1}\left(P-C{\dot{\alpha }}_{a}-G\right) \end{array}$$

The fuzzy approximation is widely recognized as an effective technique to estimate the nonlinear and uncertainty of the system in robot control, owing to its strong capability for approximations and fault tolerance (Zhao et al., 2023). In this article, the problem of time-varying dynamics and unknown friction in TAB can be effectively solved by constructing a fuzzy system to approximate the intricate nonlinear dynamics and utilizing a model-free sliding mode control scheme.

*Lemma 1*. The nonlinear dynamics *P* can be approximated by the constructed fuzzy system with reasonable errors, it is expressed as the following continuous equation (Wang, 1994).


(13) $$\begin{array}{l} P={\theta^{*}}^{\text{T}}\phi \left(\xi \right)+\varepsilon \end{array}$$

(14) $$\begin{array}{l} |\varepsilon |\leq \varepsilon_{U} \end{array}$$

where θ^*^ represents adaptive weight vector, ϕ(ξ) is the fuzzy basic function, ε is the approximation error, ε_*U*_ represents the upper boundary of fuzzy approximation errors. Define input states of fuzzy system as $x={\left[\alpha ;\dot{\alpha }\right]}^{\text{T}}$, then fuzzy basic function ϕ(ξ) is constructed as follow:


(15) $$\begin{array}{l} \phi \left(\xi \right)=\phi_{l_{1}l_{2}}\left(x\right)=\frac{\overset{2}{\underset{i=1}{\prod }}\mu_{A_{i}^{l_{i}}}\left(x_{i}\right)}{\overset{l_{M}}{\underset{l_{1}=1}{\sum }}\overset{l_{N}}{\underset{l_{2}=1}{\sum }}\left(\overset{2}{\underset{i=1}{\prod }}\mu_{A_{i}^{l_{i}}}\left(x_{i}\right)\right)} \end{array}$$

where *l*_*i*_(*i* = 1, 2) represents the number of membership, *l*_*M*_ and *l*_*N*_ represent the maximum number and the minimum number of membership, respectively, $A_{i}^{l_{i}}$ is Fuzzy set of input variables *x*_*i*_, $\mu_{A_{i}^{l_{i}}}\left(x_{i}\right)$ represents the membership function of input variables *x*_*i*_(*i* = 1, 2).

**Figure 3:**
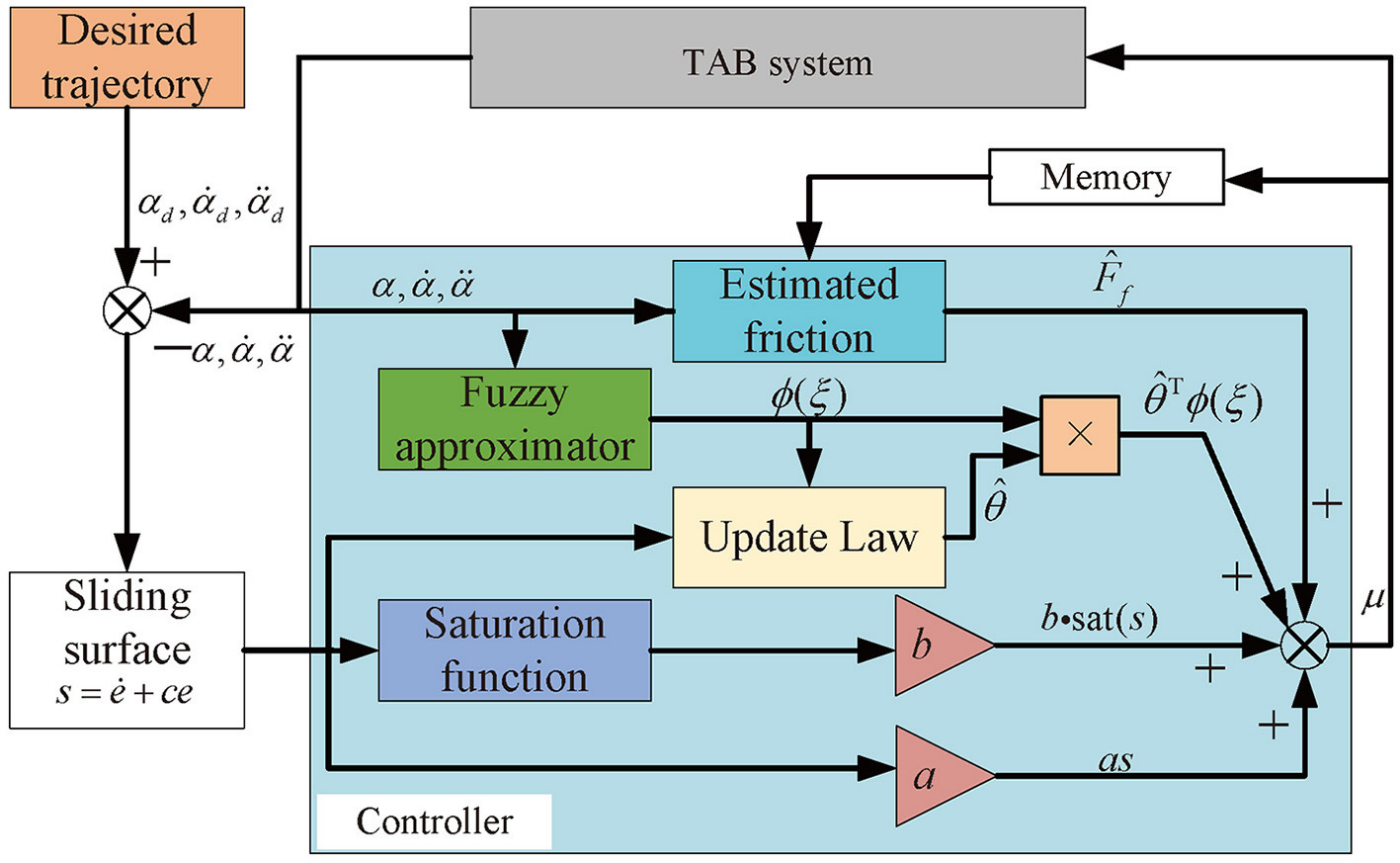
The block diagram of proposed controller.

According to the former description of the friction between tendon and sheath, the sampling time can be selected small enough to reduce the estimation errors to ensure the validity of the method (Elmali and Olgac, 1996; Wu et al., 2019). The unknown friction is estimated by the following equation:


(16) $$\begin{array}{l} {\hat{F}}_{f}=\mu_{t-T}-M\ddot{\alpha }-C\dot{\alpha }-G \end{array}$$

where ${\hat{F}}_{f}$ represents the estimated friction, μ_*t*−*T*_ is control input in the previous time step. The estimation errors ${\tilde{F}}_{f}$ are described as ${\tilde{F}}_{f}=F_{f}-{\hat{F}}_{f}$. As a result, it is reasonable to assume that the estimated errors are bounded, and the boundary can be given as follows:


(17) $$\begin{array}{l} |{\tilde{F}}_{f}|<\rho \end{array}$$

where ρ is a positive constant representing the upper boundary. In order to solve the chattering problem, the saturated function sat(*s*) and the control law are elaborately designed as follows respectively:


(18) $$\begin{array}{l} \text{sat}\left(\text{s}\right)=\{\begin{array}{cc} 1 & s>\text{Δ}\\ s/\text{Δ} & |s|\leq \text{Δ}\\ -1 & s<-\text{Δ} \end{array} \end{array}$$

(19) $$\begin{array}{l} \mu ={\hat{\theta }}^{\text{T}}\phi \left(\xi \right)+{\hat{F}}_{f}+a\cdot s+b\cdot \text{sat}\left(s\right) \end{array}$$

where $\hat{\theta }$ is the approximation value of θ^*^, *a* and *b* are two positive constants. And the approximation value $\hat{\theta }$ will be estimated by the following update law:


(20) $$\begin{array}{l} \dot{\hat{\theta }}=L\phi \left(\xi \right)s \end{array}$$

where *L* is a known positive constant. By utilizing the designed controller 19 with update law 20, time-varying dynamics and unknown friction will be compensated in real-time. The overall block diagram of the proposed strategy is shown in Figure 3.

## 4 Closed-loop stability analysis

TAB system with the proposed control law is asymptotic stable in the Lyapunov sense. The Lyapunov stability criteria was used to verify the closed-loop stability of the TAB system under the proposed adaptive fuzzy robust control strategy.

Proof. A Lyapunov candidate function is adopted as follows:


(21) $$\begin{array}{l} V=\frac{1}{2}\left(Ms^{2}+L^{-1}{\tilde{\theta }}^{\text{T}}\tilde{\theta }\right) \end{array}$$

where the errors between the ideal value and approximation are represented as $\tilde{\theta }=\theta^{*}-\hat{\theta }$. Differentiating Equation 21 and substituting Equations 10 and 11, for the ease of derivation, the auxiliary terms are added to the last two terms, $\dot{V}$ can be arranged as:


(22) $$\begin{array}{l} \dot{V}=Ms\dot{s}+\frac{1}{2}\dot{M}s^{2}-L^{-1}{\tilde{\theta }}^{\text{T}}\dot{\hat{\theta }}\\ \;=Ms\left\{{\ddot{\alpha }}_{a}-M^{-1}\left(\mu -C\dot{\alpha }-G-F_{f}\right)\right\}\\ \;-L^{-1}{\tilde{\theta }}^{\text{T}}\dot{\hat{\theta }}+\frac{1}{2}\dot{M}s^{2}-Cs^{2}+Cs^{2} \end{array}$$

According to the designed controller, based on Equation 16, by inserting Equations 19 into 22 it can be indicated as follows:


(23) $$\begin{array}{l} \dot{V}=Ms\{{\ddot{\alpha }}_{a}-M^{-1}[{\hat{\theta }}^{\text{T}}\phi \left(\xi \right)+a\cdot s+b\cdot \text{sat}\left(s\right)-C\dot{\alpha }-G\\ \;+{\hat{F}}_{f}-F_{f}]\}-L^{-1}{\tilde{\theta }}^{\text{T}}\dot{\hat{\theta }}+\frac{1}{2}\dot{M}s^{2}-Cs^{2}+Cs^{2} \end{array}$$

Then, for eliminating time-varying model parameters *M* and *G*, according to Equations 13–15, substitute Equations 12 and 13 for $\ddot{\alpha }$ and *P* respectively, Equation 23 can be re-expressed as:


(24) $$\begin{array}{l} \dot{V}=Ms\{M^{-1}\left(P-C{\dot{\alpha }}_{a}-G\right)-M^{-1}[{\hat{\theta }}^{\text{T}}\phi \left(\xi \right)+a\cdot s+b\cdot \text{sat}\left(s\right)\\ \;-C\dot{\alpha }-G-{\tilde{F}}_{f}]\}-L^{-1}{\tilde{\theta }}^{\text{T}}\dot{\hat{\theta }}+\frac{1}{2}\dot{M}s^{2}-Cs^{2}+Cs^{2}\\ \;=Ms\{M^{-1}\left[{\theta^{*}}^{\text{T}}\phi \left(\xi \right)+\varepsilon -C{\dot{\alpha }}_{a}-G\right]-M^{-1}[{\hat{\theta }}^{\text{T}}\phi \left(\xi \right)+a\cdot s\\ \;+b\cdot \text{sat}\left(s\right)-C\dot{\alpha }-G-{\tilde{F}}_{f}]\}-L^{-1}{\tilde{\theta }}^{\text{T}}\dot{\hat{\theta }}+\frac{1}{2}\dot{M}s^{2}-Cs^{2}+Cs^{2}\\ \;=s[{\theta^{*}}^{\text{T}}\phi \left(\xi \right)-{\hat{\theta }}^{\text{T}}\phi \left(\xi \right)+\varepsilon -C\left({\dot{\alpha }}_{a}-\dot{\alpha }\right)-a\cdot s\\ \;-b\cdot \text{sat}\left(s\right)+{\tilde{F}}_{f}]-L^{-1}{\tilde{\theta }}^{\text{T}}\dot{\hat{\theta }}+\frac{1}{2}\dot{M}s^{2}-Cs^{2}+Cs^{2} \end{array}$$

Further simplification, by combining Equations 8 and the definition of $\tilde{\theta }$ to Equation 24, the added positive auxiliary term *Cs*^2^ is offset, $\dot{V}$ can be derived as:


(25) $$\begin{array}{l} \dot{V}=s\left[{\tilde{\theta }}^{\text{T}}\phi \left(\xi \right)+\varepsilon -Cs-a\cdot s-b\cdot \text{sat}\left(s\right)+{\tilde{F}}_{f}\right]-L^{-1}{\tilde{\theta }}^{\text{T}}\dot{\hat{\theta }}\\ +\frac{1}{2}\dot{M}s^{2}-Cs^{2}+Cs^{2}\\ \;=s\left[\varepsilon -Cs-a\cdot s-b\cdot \text{sat}\left(s\right)+{\tilde{F}}_{f}\right]+s{\tilde{\theta }}^{\text{T}}\phi \left(\xi \right)-L^{-1}{\tilde{\theta }}^{\text{T}}\dot{\hat{\theta }}\\ +\frac{1}{2}\dot{M}s^{2}-Cs^{2}+Cs^{2}\\ \;=s\left[\varepsilon -b\cdot \text{sat}\left(s\right)+{\tilde{F}}_{f}\right]-\left(C+a\right)s^{2}+s{\tilde{\theta }}^{\text{T}}\phi \left(\xi \right)-L^{-1}{\tilde{\theta }}^{\text{T}}\dot{\hat{\theta }}\\ +\frac{1}{2}\dot{M}s^{2}-Cs^{2}+Cs^{2}\\ \;=s\left[\varepsilon -b\cdot \text{sat}\left(s\right)+{\tilde{F}}_{f}\right]-a\cdot s^{2}+s{\tilde{\theta }}^{\text{T}}\phi \left(\xi \right)-L^{-1}{\tilde{\theta }}^{\text{T}}\dot{\hat{\theta }}\\ \;+\frac{1}{2}\dot{M}s^{2}-Cs^{2} \end{array}$$

After that, by substituting update law Equation 20, and according to *Property 1 and Property 2*, i.e. Equations 3 and 4 the last four terms will be eliminated, and Equation (25) is deduced as:


(26) $$\begin{array}{l} \dot{V}=s\left[\varepsilon -b\cdot \text{sat}\left(s\right)+{\tilde{F}}_{f}\right]-a\cdot s^{2} \end{array}$$

Finally, *a* is a positive constant, and according to Equations 17, 18, Equation 26 can be expressed as follows:


(27) $$\begin{array}{l} \dot{V}\leq s\left[\varepsilon -b\cdot \text{sat}\left(s\right)+{\tilde{F}}_{f}\right]\\ \;\leq |s|\left[|\varepsilon |+|{\tilde{F}}_{f}|-b\cdot \text{sat}\left(\left|s\right|\right)\right]\\ \;\leq |s|\left(|\varepsilon |+\rho -b\right) \end{array}$$

If the positive gain *b* satisfies


(28) $$\begin{array}{l} b\geq |\varepsilon |+\rho \end{array}$$

By inserting Equations 28 into 27, $\dot{V}$ is obtained as


(29) $$\begin{array}{l} \dot{V}\leq 0 \end{array}$$

Equations 21 and 29 indicate that the chosen Lyapunov candidate *V* is positive definite, and $\dot{V}$ is negative definite. As a result, the asymptotic stability of the TAB system under the proposed control law has been proved. The angle-tracking errors gradually converge to 0 and approach the sliding surface (i.e., s = 0) in finite time.     □

## 5 Simulation and analysis

In this section, simulation experiments and results are provided to validate the performance of the proposed control scheme. Firstly, three different tracking trajectories are designed to evaluate the tracking performance of the control scheme. Then, experiments on varying-friction with and anti-disturbance experiments are conducted to compare the performance and robustness of the proposed scheme with the standard PID controller, sliding mode-PI (SMPI) controller, and linear quadratic regulator (LQR) controller. The reason for choosing PID-based methods and LQR as the controllers for comparison is because they have been widely applied and demonstrated good performance in the field of robotic flexible endoscope (Jiang et al., 2020; Kong et al., 2023). It should be pointed out that the three comparative control methods have been modified according to the dynamics model of this paper, and the control gains have been correspondingly adjusted by trial and error to guarantee tracking accuracy. The three controllers and their elaborately tuned control gains are adopted as follows respectively:

1. Standard PID controller
  (30) $$\begin{array}{l} \mu =K_{p}e+K_{i}\int e\text{d}t+K_{d}\dot{e} \end{array}$$
  where *K*_*p*_, *K*_*i*_, *K*_*d*_ are positive gains. After appropriate optimization adjustments, they are selected as *K*_*p*_ = 1000, *K*_*i*_ = 3000, *K*_*d*_ = 200.
2. SMPI controller
  (31) $$\begin{array}{l} s=\dot{e}+ce\\ \mu =K_{sp}s+K_{si}\int s\text{d}t \end{array}$$
  where *e* = α_*d*_ − α, *c*, *K*_*sp*_ and *K*_*si*_ are positive gains. They are chosen as *c* = 1.5, *K*_*sp*_ = 3, *K*_*si*_ = 3000.
3. LQR controller
  (32) $$\begin{array}{l} J=\int_{0}^{\infty }e^{\text{T}}Qe+\mu^{\text{T}}R\mu \text{d}t\\ \mu =-Ke \end{array}$$
  where ***e*** = [*e, ė*]^T^, ***Q*** and ***R*** are weighting matrix. They are selected as ***Q*** = diag{1, 20}, ***R*** = diag{0.01}.

### 5.1 **Tracking experiments in different cases**

To validate the effectiveness of the proposed control algorithm, a series of numerical simulations were carried out in a MATLAB/Simulink environment. To ensure the fidelity of the simulation, parameters in the dynamics model were selected to align with the actual material parameters of the real TAB system, as presented in previous work (Wang et al., 2023).


(33) $$\begin{array}{l} m=0.01\text{kg},E=500\text{Pa},D=0.003\text{m}\\ L_{f}=0.15\text{m},\;\alpha \left(0\right)=5\deg \end{array}$$

Then, to achieve satisfactory tracking performance, the control gains in Equations 19 and 20 are elaborately tuned as:


(34) $$\begin{array}{l} a=5,b=2,c=2,L=2 \end{array}$$

Considering both computational accuracy and computational cost, the membership function of each state is set to 5 fuzzy subsets corresponding to its value range. Then, the fuzzy logic system with 2 input states α and $\dot{\alpha }$ can produce 25 fuzzy rules. Based on the operational constraints of α and $\dot{\alpha }$ applied in real robotic flexible endoscope, the Gaussian membership functions are selected as follows respectively by trial and error.


(35) $$\begin{array}{l} \{\begin{array}{c} f\left(\alpha ,\sigma_{1},v_{i}\right)=e^{-\frac{{\left(\alpha -v_{i}\right)}^{2}}{2{\sigma_{1}}^{2}}}\\ f\left(\dot{\alpha },\sigma_{2},w_{i}\right)=e^{-\frac{{\left(\dot{\alpha }-w_{i}\right)}^{2}}{2{\sigma_{2}}^{2}}} \end{array} \end{array}$$

where σ_1_, σ_2_, *v*_*i*_ and *w*_*i*_ (*i* = 1, 2, 3, 4, 5) are prechosen coefficients. The values of them are selected as $\sigma_{1}=1.39,\sigma_{2}=0.93,v_{i}=\left[\frac{5\left(9-\pi \right)}{2},\frac{5\left(18-\pi \right)}{4},22.5,\frac{5\left(18+\pi \right)}{4},\frac{5\left(9+\pi \right)}{2}\right],w_{i}=\left[\frac{5\pi }{3},\frac{5\pi }{6},0,-\frac{5\pi }{6},-\frac{5\pi }{3}\right]$.

*Remark 1*. In this article, the parameters in the control law are tuned by trial and error to guarantee optimal performance. Fine-tuning *a* and *c* will reduce response time, *L* is closely associated with the steady-state error of the system, and proper *b* can effectively prevent system oscillations or overshoot. Parameters in fuzzy logic are empirically tuned by trial and error to ensure the accuracy of approximation. σ_1_ and σ_2_ influence the shape of the membership functions, thereby affecting the results of fuzzy inference. *v*_*i*_ and *w*_*i*_ are empirically partitioned into equal intervals based on the operational range.

To verify the tracking performance of the proposed controller, three different tracking cases are chosen as follows:

1) Case 1: (step trajectory) The amplitude of the step trajectory is set as 20 deg.

2) Case 2: (sine trajectory) The frequency, the initial phase, the amplitude, and the offset of the desired sine trajectory are set as π rad/sec, 0 deg, 5 deg, and 15 deg, respectively.


(36) $$\begin{array}{l} y_{d}\left(t\right)=5\sin\left(t\right)+15 \end{array}$$

3) Case 3: (triangular-wave trajectory) The frequency, amplitude, and offset of the desired triangular-wave trajectory are set as 0.1 Hz, 7.5 deg, and 17.5 deg, respectively.

**Figure 4:**
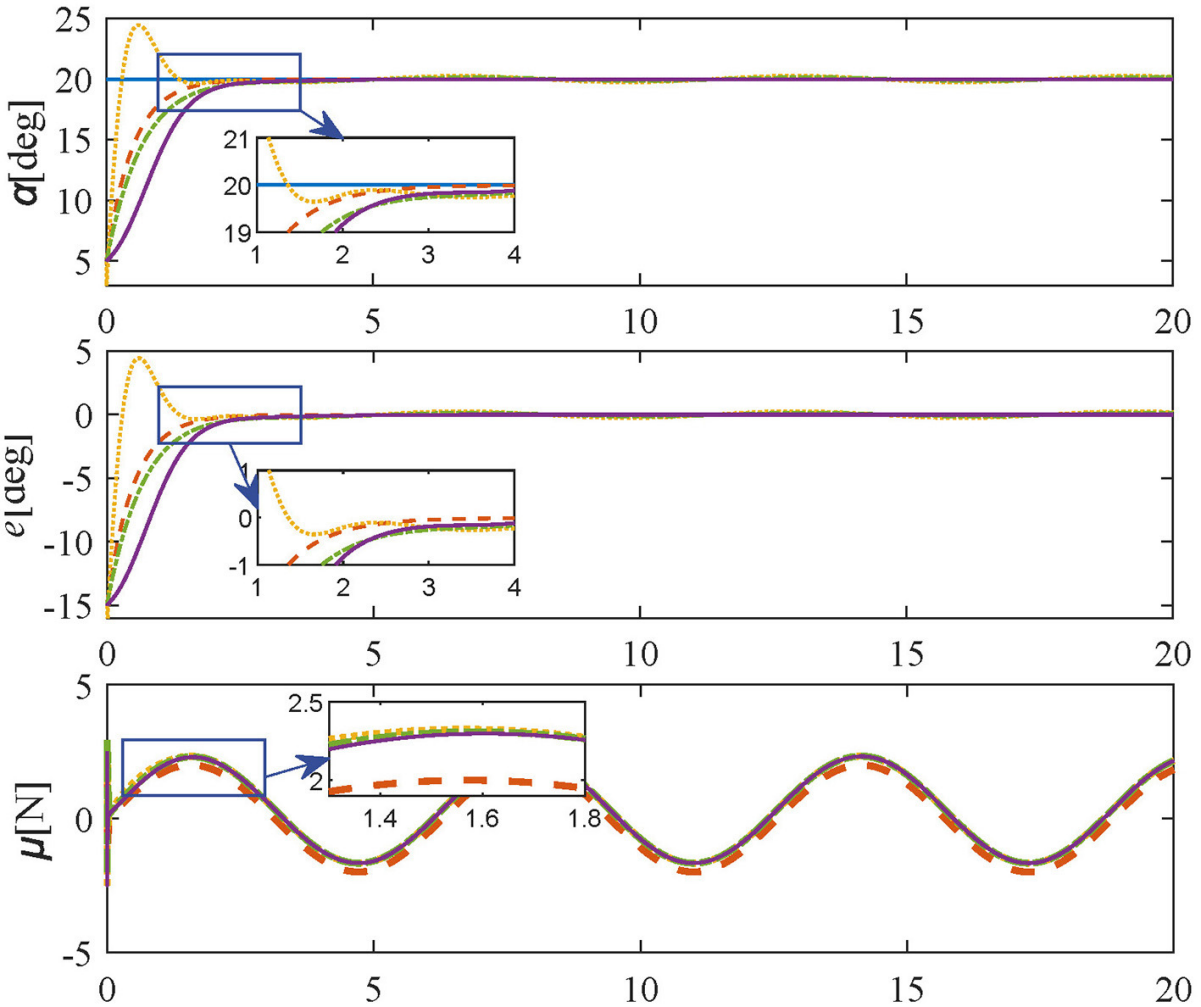
Simulation results in **Tracking experiment:** Case 1 (reference values-blue solid line; proposed controller-orange dashed line; PID controller-yellow dotted line; SMPI controller-green dash-dot line; LQR controller-purple solid line).

In **Tracking experiments: Case 1**, the parameters of the proposed method are set as in Equations 33 and 34. Also, the initial and target angles are set to 5 deg and 20 deg respectively. The simulation results of Tracking experiments: Case 1 are shown in Figure 4, the desired angle can be reached quickly within 3 s by adopting the proposed controller without overshoot or oscillation, demonstrating the regulation performance of the proposed control scheme. In contrast, comparative methods, i.e. Equations 30–32 take more time to be steady and are accompanied by oscillating and residual errors.

**Figure 5:**
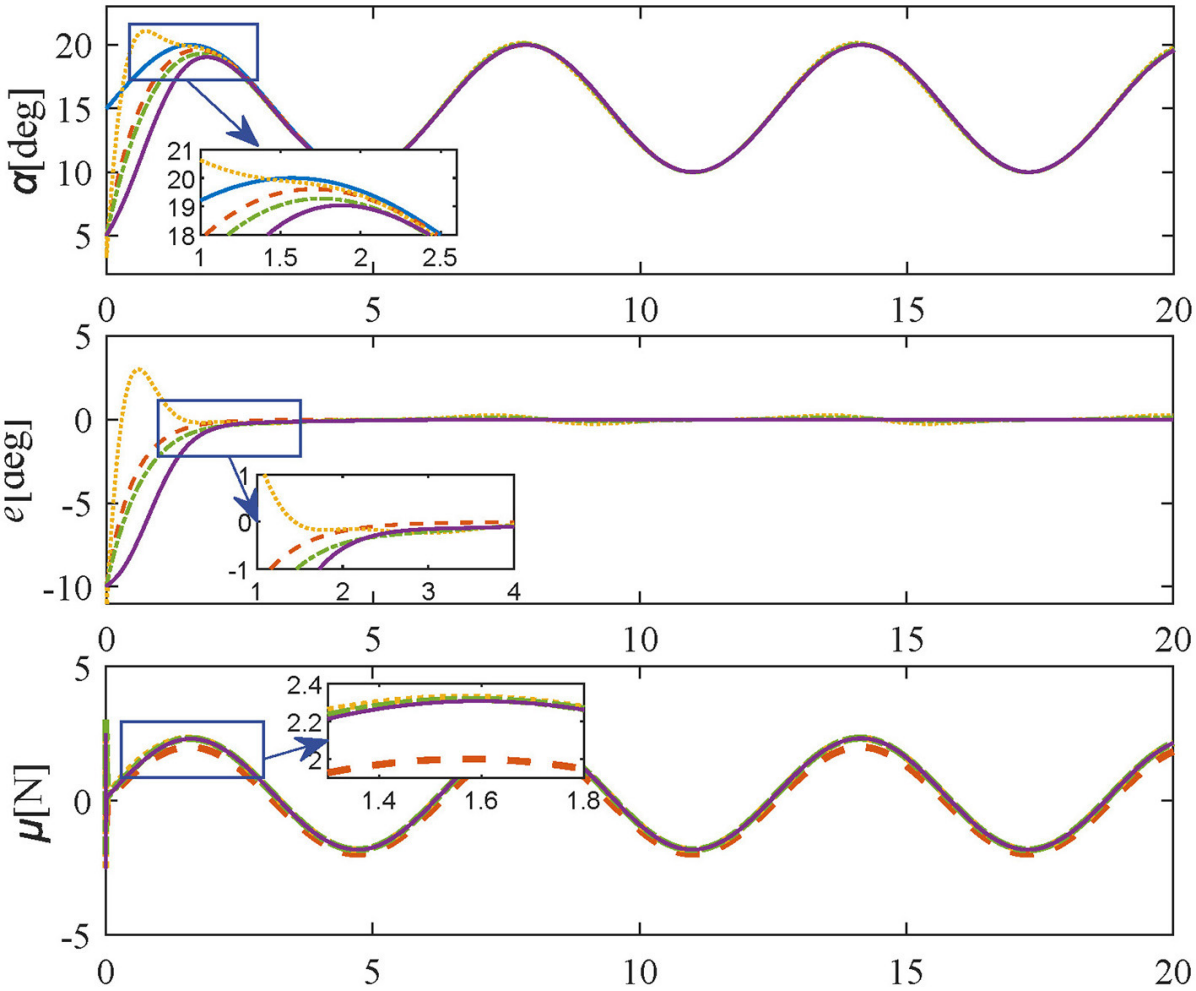
Simulation results in **Tracking experiment:** Case 2 (reference values-blue solid line; proposed controller-orange dashed line; PID controller-yellow dotted line; SMPI controller-green dash-dot line; LQR controller-purple solid line).

In **Tracking experiments: Case 2**, to further confirm the tracking performance of the proposed method, the same parameters are set as Case 1. The initial angle and desired trajectory are set to 5 deg and Equation 36, respectively. The results of Tracking experiments: Case 2 are given in Figure 5, the angle errors can be quickly converged to 0 within 3 s without overshoot or oscillation by adopting the proposed control method. In contrast, as shown in Figure 5, comparative methods take a longer time to achieve the desired trajectory, and there are obvious overshooting and oscillations. Therefore, the simulation results show that the proposed method has a faster response and smaller steady-state errors than comparative controllers.

**Figure 6:**
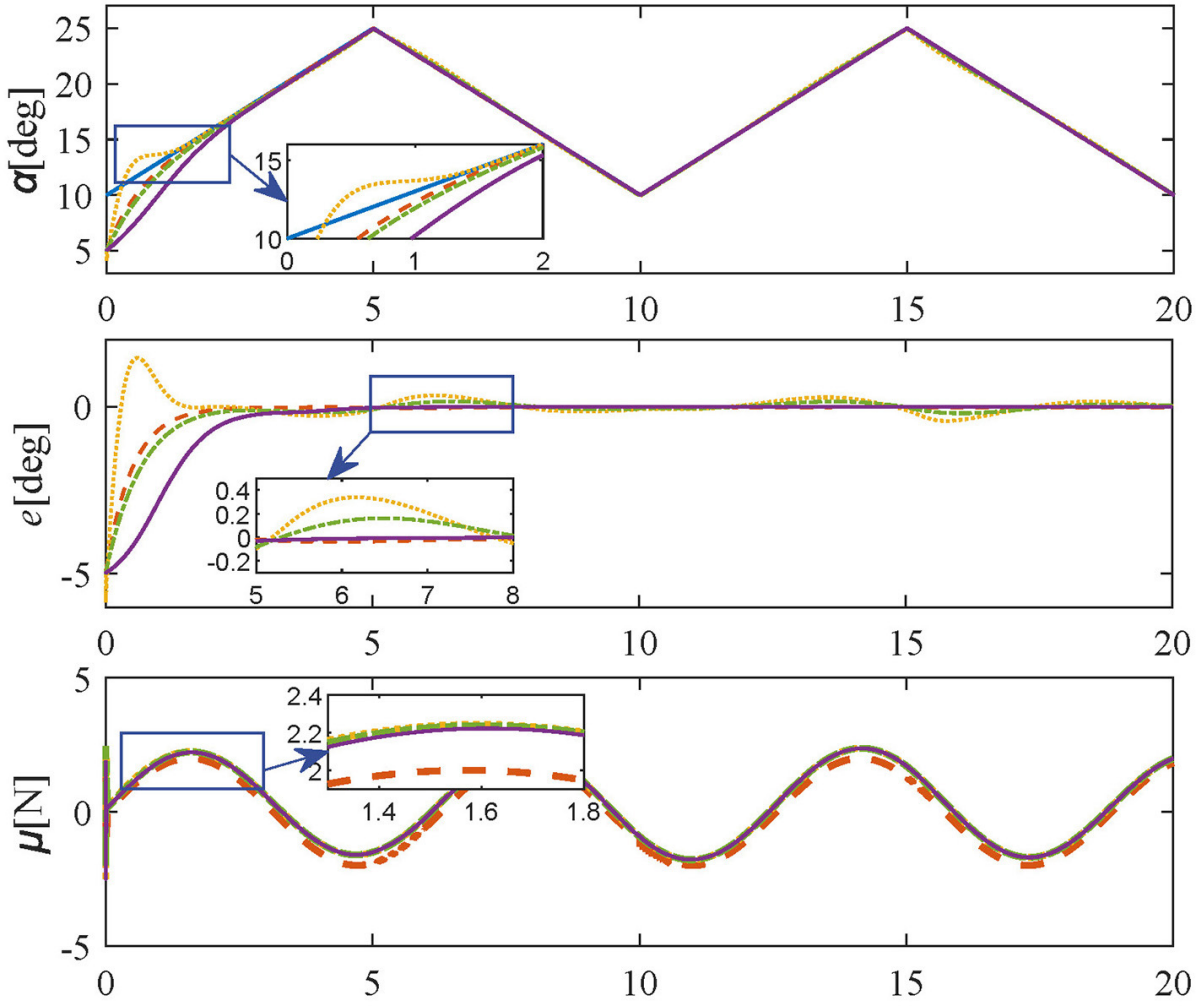
Simulation results in **Tracking experiment:** Case 3 (reference values-blue solid line; proposed controller-orange dashed line; PID controller-yellow dotted line; SMPI controller-green dash-dot line; LQR controller-purple solid line).

The simulation results of **Tracking experiments: Case 3** are shown in Figure 6, the bending-tip can reach the desired trajectory within 3 s by using the proposed control method. For comparative methods, the response time is longer. Besides, oscillations still exist for comparative methods at 5, 10, and 15 s. In contrast, the proposed method guarantees regulation stability without any residual errors. Therefore, the simulation results of three different cases in tracking experiments show that the proposed method has satisfactory tracking performance and acceptable steady response.

### 5.2 **Varying-friction experiment**

In order to test the dynamic adaptability of the proposed controller, in this experiment, the friction force is set as a time-varying signal with constant amplitude and varying offset for simulating the switching of working conditions. Three bounded sets of unidentified friction, each representing different working conditions due to different ranges were employed. It is worth mentioning that the form of friction in this experiment is just selected as an example, the controller proposed in this paper only requires the friction force to be bounded without a specific model, which is a contribution of this paper. The specific form of friction force is as follows:


(37) $$\begin{array}{l} F_{f}=\{\begin{array}{cc} \sin t & 0\leq t<6.28\\ \sin\left(t+\pi /2\right)-1 & 6.28\leq t<12.56\\ \sin\left(t-\pi /2\right)+1 & 12.56\leq t\leq 20 \end{array} \end{array}$$

**Figure 7:**
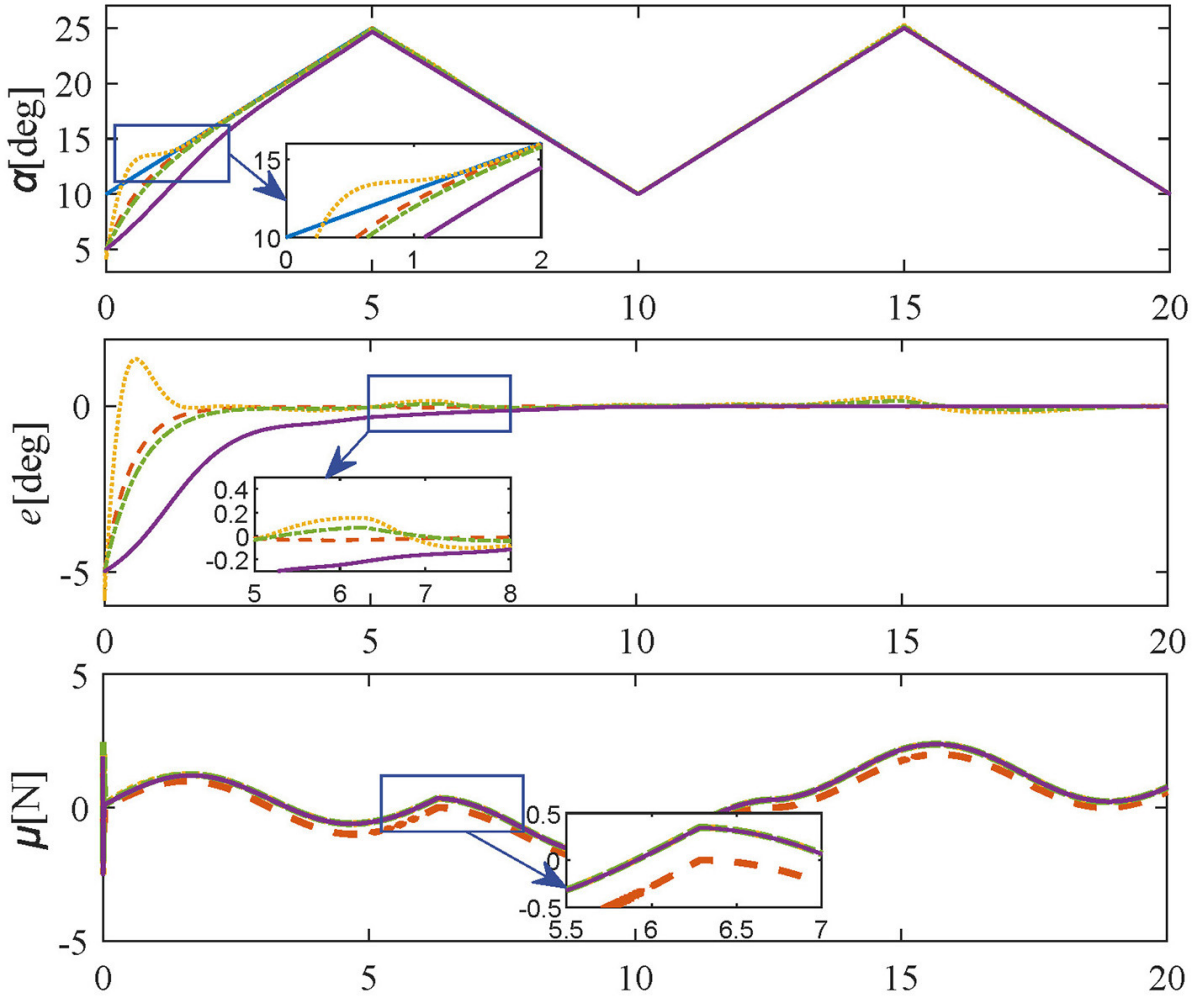
Simulation results in **Varying-friction experiment** (reference values-blue solid line; proposed controller-orange dashed line; PID controller-yellow dotted line; SMPI controller-green dash-dot line; LQR controller-purple solid line).

The simulation results of **Varying-friction experiment** are shown in Figure 7, the same parameters are set as in Tracking experiments: Case 3. As shown in Figure 7, the proposed method still achieves the desired trajectory rapidly and accurately without steady-state errors. In contrast, the PID controller and SMPI controller have obvious oscillations near 6.28 s and 12.56 s, and the LQR controller has a much slower transient response. In consequence, the proposed method can effectively cope with varying friction Equation 37 under different working conditions.

### 5.3 Anti-disturbance experiment

**Figure 8:**
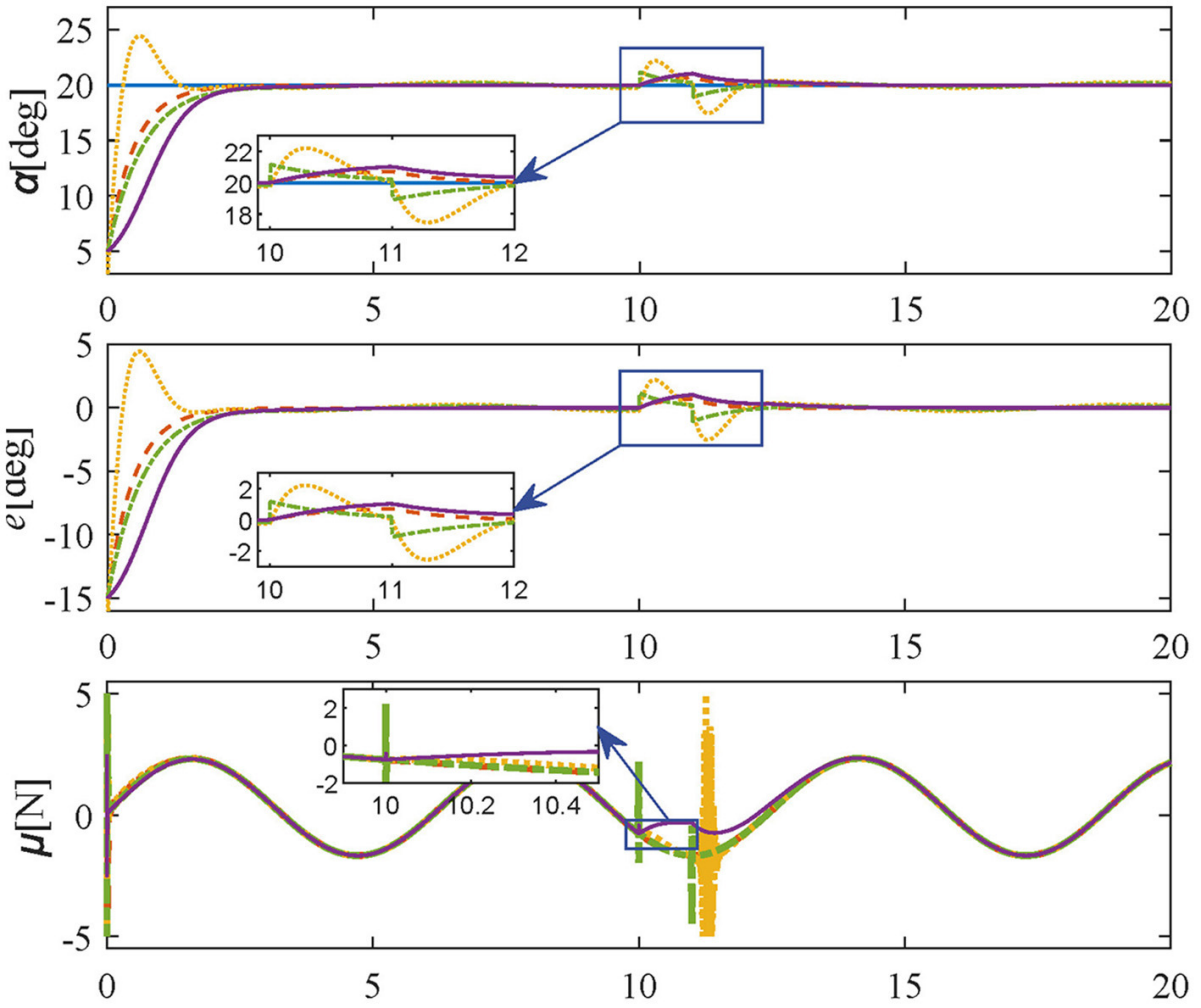
Simulation results in **Anti-disturbance experiment** (reference values-blue solid line; proposed controller-orange dashed line; PID controller-yellow dotted line; SMPI controller-green dash-dot line; LQR controller-purple solid line).

To verify the robustness of the controller suffering episodic disturbances in the unstructured environment, a constant external force of 1N lasting 1 second is added at the 10th second on the basis of Tracking experiments: Case 1, and the simulation results of **Anti-disturbance experiment** are shown in Figure 8. The angle errors generated by external disturbances are less than 1 deg under the proposed controller, which returns to zero in 2 s. In contrast, the same disturbances under comparative methods excite larger amplitudes (LQR) and sharper force fluctuations (PID, SMPI). In summary, the proposed method is non-sensitive to disturbances and fast-tracking of the desired trajectory without steady-state errors. As a result, it is possible to conclude with certainty that the proposed controller has both excellent tracking performance and satisfactory robustness.

*Remark 2*. Although the method proposed in this paper achieves effective tracking and compensation of unknown bounded friction, there are still some limitations. One concern about the proposed controller is that the setting of fuzzy logic membership functions and fuzzy rules relies on empirical knowledge and requires considerable effort to tune for satisfactory performance. It is worth mentioning that reinforcement learning has demonstrated significant performance in finding optimal control policy in recent years (Goharimanesh et al., 2020). As a consequence, fuzzy control based on reinforcement learning is poised to become a further improvement for our method in the future.

## 6 Conclusions

Based on the Lagrangian dynamics model for the TAB systems, this paper proposed a robust controller for tracking tasks. Specifically, the nonlinear friction with consideration of its boundary is described according to the dynamics model of TAB. Then, the fuzzy logic system is used to achieve the estimation of nonlinear time-varying dynamics. At last, a sliding mode control method was designed, which achieved effective tracking performance and compensation of the unknown boundary friction. Lyapunov stability criteria was also utilized to prove the asymptotic stability of the proposed controller. Simulations are also carried out to validate the efficiency of the proposed method. The proposed method is model-free control and has no strict requirement for the dynamics model and friction model. It is proved that advanced tracking performance and real-time response can be guaranteed under the presence of unknown bounded nonlinear friction and time-varying nonlinear dynamics. In future research, we will further investigate the universal applicability of the proposed method in addressing unknown friction and its practical utilization in experiments involving flexible endoscope robots.

## Data availability statement

The raw data supporting the conclusions of this article will be made available by the authors, without undue reservation.

## Author contributions

FR: Data curation, Methodology, Software, Writing – original draft, Conceptualization. XW: Funding acquisition, Project administration, Supervision, Writing – review & editing. NY: Funding acquisition, Writing – review & editing. JH: Funding acquisition, Writing – review & editing.
